# The effect of combining HIIT and dry-land training on strength, technique, and 100-m butterfly swimming performance in age-group swimmers: a randomized controlled trial

**DOI:** 10.5114/biolsport.2023.110747

**Published:** 2022-01-03

**Authors:** Sofiene Amara, Raouf Hammami, Rodrigo Zacca, Jorge Mota, Yassine Negra, Sabri Gaied Chortane

**Affiliations:** 1 Higher Institute of Sport and Physical Education of Ksar-Said, University of La Manouba, Tunis, Tunisia; 2 Research Unit (UR17JS01) Sports Performance, Health & Society, Higher Institute of Sport and Physical Education of Ksar Saîd, Universite de la Manouba, Tunis 2010, Tunisia; 3 Research Laboratory: “Education, Motor Skills, Sports and Health” (LR19JS01), Higher Institute of Sport and Physical Education of Sfax, University of Sfax, Sfax 3029, Tunisia; 4 Research Center in Physical Activity, Health and Leisure (CIAFEL), Faculty of Sports, University of Porto (FADEUP), Porto 4200-450, Portugal; 5 Laboratory for Integrative and Translational Research in Population Health (ITR), Porto 4050-600, Portugal; 6 Laboratory of Cardio-Circulatory, Respiratory, Metabolic and Hormonal Adaptations to Muscular Exercise, Faculty of Medicine Ibn El Jazzar, Sousse 4002, Tunisia

**Keywords:** Exercise, Aquatic locomotion, Training and testing, Combined training, High intensity interval training

## Abstract

Combined interventions of pool-based and dry-land workouts are a common practice in swimming training. However, the effects on strength, technique and swimming performance are still not clear. Through a randomized controlled trial study, we investigated the effect of combining high intensity interval training (HIIT) and maximum strength training (MST) on strength, technique and 100-m butterfly swimming performance. Competitive age-group swimmers (N = 22, males) were randomly divided into two groups. The experimental group (EG: 14.1 ± 0.3 years old) performed 8 weeks of combined short-moderate HIIT and MST. The control group (CG: 14.5 ± 0.3 years old) subjects performed their usual training. Muscular strength, technique and swimming performance were evaluated before and after 8 weeks. Substantial improvements were observed in maximum muscle strength (mean diff: 22–28%; p < 0.001; d = 3.25–3.61), technique (p < 0.05; d = 0.98–1.96) and 100-m butterfly swimming performance (3.5%; p = 0.001; d = 1.81) when combining HIIT and MST during 8 weeks. Combining short-moderate HIIT and MST during 8 weeks can enhance maximum muscular strength, technique, and 100-m butterfly swimming performance. Coaches should adjust training programmes accordingly since it could yield important differences in swimming performance during competitions.

## INTRODUCTION

Performance in competitive swimming is a multifactorial phenomenon, i.e., it is determined by a relatively large number of factors (e.g., energetics, technique, anthropometrics) [1–3]. When it comes to improving swimming performance, it is well reported that dry-land workouts including maximum strength training (MST) can improve it [4–5]. Likewise, high intensity interval training (HIIT) workouts can enhance swimming performance [6]. Most swimming-related studies verified the effects of MST and HIIT on swimming performance in an independent manner, and generally in front crawl swimming. Although relevant, it is also necessary to observe this phenomenon in a randomized approach, i.e., by examining the combined effect of MST and HIIT on strength, technique and swimming performance, and also particularly in other swimming techniques (e.g., butterfly).

Combined dry-land workouts and HIIT have been applied in some sports [7–8]. Combining strength workouts (2–6 sets of parallel squats at 80% of one-repetition maximum, 1-RM) and HIIT (8–24 “Tabata intervals” at ˜150% of maximal oxygen uptake, O_2max_) for 6 weeks could improve O_2max_ and performance of parallel squats (4 ± 3% and 14 ± 10%, respectively) in highly skilled ice hockey and rugby players [7, 9]. Aspenes et al. [9] suggested that the 1-RM and 400-m front crawl swim performance improved after 11 weeks (16.87% and 1.38%, respectively) when combining HIIT and MST. Eighteen sessions of HIIT over 2 weeks were enough to improve 750-m swim (4%) and 20-km cycling time (8%), and peak oxygen uptake (O2peak) (2%) in adolescent triathletes [10]. Sperlish et al. [6] suggested that 5 weeks of HIIT (2 sessions · week^-1^) could improve O_2max_ and performance of 2000-m and (10%, 3%, respectively) in competitive age-group swimmers. In fact, HIIT is an effective training modality for improving endurance quality in numerous sports [6, 10]. HIIT workouts can be categorized based on interval work-bout duration: short HIIT if work bouts are under 30 s, medium HIIT if 30 s to 2 min, and long HIIT if 2 to 4 min [11–12]. However, information on technique adaptation after combining MST and HIIT remains scarce in age-group swimmers, particularly other swimming techniques.

The metabolic power (energy expended per unit of time) is very important in aquatic locomotion [1, 5, 13]. An increase in metabolic power with swimming speed (*v*) reflects the increase in (total) mechanical power output that muscles need to provide while sustaining that *v* [1]. Additionally, maximum upper body strength (bench press) and lower body (back squat) explain ˜55–65% of the performance in the tethered swimming power, ˜50% of swim start performance and 45–62% of 50-m and 100-m front crawl swimming performance [13]. Amaro et al. [14] suggest that the MST stimulus must be characterized by an intensity greater than 80%1-RM, in 2 to 3 sets with 3 to 5 repetitions and a recovery interval between 2 and 5 min. Bench press (BP), leg extension (LE) and back squat (BS) are widely used in dry-land training protocols to improve upper and lower body strength in swimmers [15]. In the same perspective, maximum upper and lower body strength could be determined by 1-RM of bench press (1-RM BP) and 1-RM of leg extension (1-RM LE) [13, 16].

Evidence on the effect of combining strength training (only upper body) and HIIT on front crawl swimming performance is scarce [8]. This reinforces that we need more evidence to understand the effect of combining HIIT and MST (upper and lower body) on swimming performance, particularly in swimming techniques other than front crawl. Through a randomized controlled trial, we verified the effect of 8 weeks of combining HIIT and dry-land training with MST in upper and lower body on changes in muscle strength, technique and butterfly swimming performance. We hypothesized that such an approach would improve maximum muscular strength, technique, and 100-m butterfly swimming performance in competitive age-group swimmers.

## MATERIALS AND METHODS

### Study design

In this randomized controlled study, twenty-two swimmers were randomly allocated to an experimental group (EG), combining MST and HIIT, and a control group (CG), which performed habitual training. Maximum muscle strength (1-RM BP and 1-RM LE), technique (velocity, stroke rate, stroke length and stroke index) and swimming performance in 100-m butterfly swimming were measured before and after 8 weeks of both training conditions. Swimmers were familiarized during 1 week before the first testing with bench press (BP) and leg extension (LE) repetitions. In addition, swimmers were instructed not to perform any other physical training regimen in terms of speed and strength over the study period. All participants were injury free prior to the preliminary practice.

### Participants

National level age-group swimmers (N = 22, males), specialized in butterfly, volunteered to participate in the current study. They were randomly allocated to an experimental group (EG, N = 11; age: 14.1 ± 0.30 years; height: 170 ± 9.8 cm; body mass: 68.7 ± 4.3 kg) and control group (CG, N = 11; age: 14.5 ± 0.32 years; height: 171 ± 8.4 cm; body mass = 68.1 ± 3.8 kg). An a priori power analysis (G*Power 3.1.9.3) showed that a sample size of 8 was necessary to detect large effects (*d* = 1.33) using a power of 0.8 and alpha of 0.05. The swimmers are specialists in butterfly swimming, with more than 6 years of competitive experience in swimming and 3 years of strength training. Swimmers’ parents were informed about the benefits and risks of participating in the current study prior to signing an informed consent form, which was approved by the ethics board of the Higher Institute of Sport and Physical Education of Ksar Saïd, University of Manouba, Tunisia, in accordance with the Helsinki Declaration. Table 1 presents characteristics of subjects from experimental (EG) and control (GC) groups.

**TABLE 1:** Subjects characteristics (mean ± SD) from experiment (EG) and control (GC) group

	EG (n = 11)	CG (n = 11)
**Age** (yr-old)	14.1 ± 0.30	14.5 ± 0.32
**Height** (cm)	170 ± 9.8	171 ± 8.4
**Body mass** (kg)	68.74 ± 4.35	68.08 ± 3.79
**Arm span** (cm)	173 ± 10.2	173 ± 10.7
**Competitive swimming experience** (years)	6.43 ± 0.29	6.34 ± 0.25
**Dry-land experience** (years)	3.51 ± 0.24	3.50 ± 0.26

### Procedures

Pool-based training and swimming performance tests took place in a 50-m indoor pool with 27.3 and 25.8°C water and air temperatures (respectively), and 64% relative humidity. Dry-land training and strength tests were performed in a bodybuilding room.

### Training in the water

**FIG. 1:**
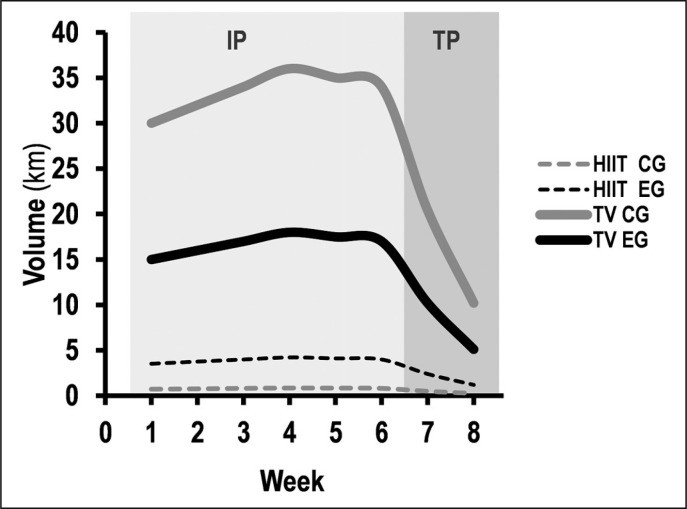
HIIT and training volume over 8 weeks of pool based training for both groups (CG and EG). IP: intervention period; TP: taper period; HIIT: high intensity interval training with intensity > 85% HRmax.

The CG subjects were invited to continue their usual programme, characterized by low intensity and high volume (Fig. 1 and Table 2). The EG was invited to perform a training programme with an increase in HIIT volume but lower total volume (Fig. 1 and Table 2) compared the CG [17–18]. The two training programmes were composed of three major training categories:

**TABLE 2:** Detailed description of 8-weeks for the Control Group (GC) and the Experimental Group (EG)

		Intervention period	Taper period	W1	W2	W3	W4	W5	W6	W7	W8
**Total Training Volume** (km)	**CG**	201.0	30.6	30.0	32.0	34.0	36.0	35.0	34.0	20.4	10.2
	**EG**	105.0	15.3	15.0	16.0	17.0	18.0	17.5	17.0	10.2	5.1

**Li-Aerobic** (km)	**CG**	187.4	28.5	28.0	29.8	31.7	33.6	32.6	31.7	19.0	9.5
	**EG**	70.9	10.8	10.6	11.3	12.0	12.7	12.4	12.0	7.2	3.6

**Hi-Aerobic** (km)	**CG**	10.4	1.4	1.3	1.4	1.5	1.6	1.5	1.5	0.9	0.5
	**EG**	5.9	0.9	0.9	0.9	1.0	1.1	1.0	1.0	0.6	0.3

**HIIT** (km)	**CG**	4.7	0.7	0.7	0.8	0.8	0.8	0.8	0.8	0.5	0.2
	**EG**	23.7	3.6	3.5	3.8	4.0	4.2	4.1	4.0	2.4	1.2

W: week; Li-Aerobic: low intensity-Aerobic with intensity < 70% HRmax; Hi-Aerobic: high intensity-Aerobic with intensity between 70 to 85% HRmax; HIIT: high intensity interval training with intensity > 85% HRmax.

- *Low-intensity aerobic (LI-Aerobic): warm-up, warm-down, technique exercises and low- to moderate-intensity training (< 70% of maximal heart rate, HR max; e.g. 20 × 100 m);*
- *High-intensity aerobic (Hi-Aerobic): High-intensity aerobic training (70–85% HR max; e.g. 5 × 400-m);*
- *High-intensity interval training (HIIT): short HIIT (volume: < 30 s; e.g. 8 × 50 m) and moderate HIIT (volume: 30 s to 2 min; e.g. 4 × 100 m); Intensity: > 85% HRmax; recovery time between repetitions: ratio 1:1; recovery between sets: 3 min; Table 2 and Fig. 1).*

The HR was recorded during the first 10 s after cessation of the bout using a Polar Team 2 (Finland) [19].

Both training programmes (CG and EG) were performed for 8 weeks (6-sessions · week^-1^):

- *Intervention Period (IP, 6 weeks): The CG performed 201 km of total training volume and 4.7 km of HIIT volume. The EG performed 105 km of total training volume and 23.6 km of HIIT volume [17–18];*
- *Taper Period (TP, 2 weeks): Reduction of training volume during the taper period was applied accordingly Mujika et al. [20]. The total training volume and volume of three major training categories (Li-Aerobic, Hi-Aerobic and HIIT) of both groups decreased by 40% in the first and 70% in the second week of TP compared to the last week of IP (week 6). The intensity (workload) remained unchanged for both groups during TP [20].*

### Dry-land training

**FIG. 2:**
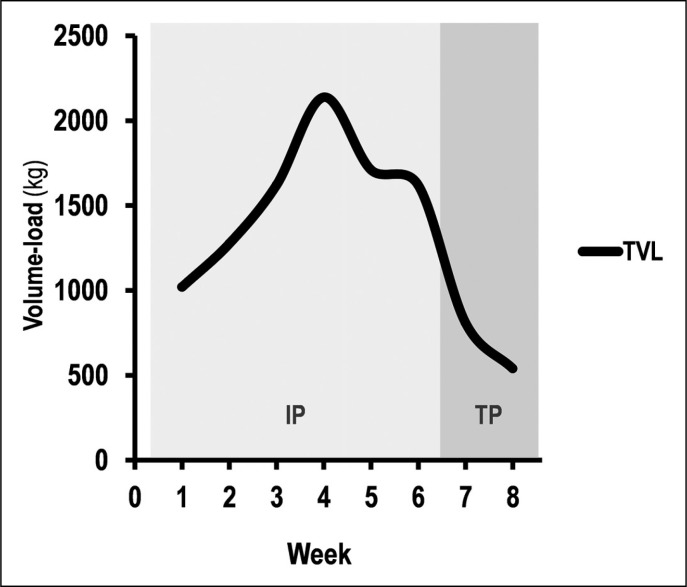
Total volume load in dry-land training over 8 weeks of maximum strength training for the experimental group. IP: intervention period; TP: taper period; TVL: total volume load with intensity between 85 and 95% 1-RM.

The dry-land programme was applied by experienced strength and conditioning coaches. The CG was invited to continue the usual dry-land training, characterized by general core strength (push-up, abdominal exercises, squat). The EG was invited to perform a dry-land MST programme, including bench press and leg extension exercises (2–5 sets × 3–5 repetitions × 85–95% 1RM) [16, 21]. Volume-load bench press (VL BP) and leg extension (VL LE) were determined according to the following equation, i.e. by multiplying the load lifted by the repetitions completed in a given set [22]:

Volume-load = *Number of sets · Number of repetitions · % 1RM*

where the 1RM of each subject was measured at pre-test. The total volume load (TVL) was then calculated by the sum of VL BP and VL LE (Fig. 2).

The two dry-land training programmes are characterized by two periods:

- *6 weeks of IP (2 sessions · week^-1^): the EG performed 9360.9 ± 735.08 kg of TVL (4628.7 ± 366.87 kg [VL BP] + 4732.2 ± 401.49 kg [VL LE]) with intensity fixed between 85 and 95% 1-RM. The recovery time between sets and exercises ranged from 3 to 5 min. The CG performed general core strength training with volume time (VT) fixed at 75 min and intensity fixed between 70 and 85% HRmax [15–16, 21].*
- *2 weeks of TP (1 session · week^-1^): Reduction of training volume was accordingly Pritchard et al. [23]. However, for both groups, training volume decreased by 40% in the first week (EG: TVL: 810 ± 65.87 kg = 399.6 ± 31.67 kg [VL BP] + 410.4 ± 35.82 kg [VL LE]; CG: VT: 45 min) and 60% in the second week (TVL: 540 ± 43.91 kg = 266.4 ± 21.12 kg [VL BP] + 273.6 ± 23.88 kg [VL LE]; CG: VT: 30 min) in relation to the last week of the intervention period (week 6). The intensity remained unchanged in both groups during this period [23].*

### Testing procedure

All the tests were performed within two days (standardized order), with dry-land measurements being taken on the first day and pool-based measurements on the second day.

### Muscle strength

The 1-RM protocol started with a standard warm-up where all subjects were invited to cycle during 3 min in an ergocycle, followed by 5 min of general static stretching. Then, subjects performed one set of 8–10 repetitions at 50% of 1-RM (estimated) and one set of 3 repetitions at 70% of 1-RM for both exercises (bench press, BP; and leg extension, LE). During the test, the load was gradually increased (10 to 20%) while the number of repetitions decreased (2 to 3), with 2 to 4 min of recovery. Then, a small increase in the load (5%), with 2 to 4 min of recovery, was performed to establish the 1-RM for BP and LE. The test ended when the subjects failed to complete the correct movement of the leg extension and of the bench press, and the last successful attempt represents the 1-RM. To maintain a good command of the bench press execution technique, the Smith machine was used during the 1-RM BP test, while the leg extension machine was used during the 1-RM LE test [24]. Two coaches specializing in strength and conditioning controlled all the tests.

### Swimming performance

All swimmers completed an 800-m warm-up (600-m low- to moderate-intensity swimming [< 70% of HR max] and 200-m progressive sprint) prior to completing two maximum trials of 100-m butterfly (with 30 min of rest between trials) [25]. Two experienced timekeepers measured the time of each trial with a stopwatch (SEIKO S120-4030, Japan). The best performance value between both trials was used. Intraclass correlation coefficient (ICC) of the two trials for Pre- and Post-test were 0.99 (p < 0.001) and 0.98 (p < 0.001), respectively.

### Technique variables

A surface video camera, Sony SNC VB 603 (50 Hz, full HD, 1080 p) was used to assess technique variables. The video camera was positioned laterally (5 m above the water and 10 m away from the edge of the pool), within two points to exclude the influence of the turning phase (1^st^ 50 m: between 20 and 40 m, 2^nd^ 50 m: between 70 and 80 m). Data were analysed with Kinovea software (Kinovea 0.9.1; Joan Charmant & Contrib., kinovea.org) [25–26]. The *v* of each 10 m was measured from the time taken to cover the middle 10 m of the two distances (*v* = d · (t10-m)^-1^, were t10-m = time for the 10 m and d = 10 m). Stroke rate (SR) was calculated from the time taken to complete three consecutive stroke cycles. Stroke length (SL) was computed from the ratio between *v* and corresponding SR. Stroke index (SI) was assessed by multiplying *v* by SL [27–29].

### Statistical analyses

The data analyses were performed using IBM SPSS Statistics for Windows, version 20.0 (IBM Corp., Armonk, N.Y., USA). The values are presented as mean ± SD. Normality was tested with the Kolmogorov-Smirnov test. The intraclass correlation coefficient (ICC) was applied to determine the reliability of the measurements [30]. Repeated measures ANOVA was used to identify the differences between pre- and post-test in the two groups (time factor). The effect size (*d*) was determined by converting partial eta-squared to Cohen’s *d* [31]. The statistical significance was set at p ≤ 0.05.

## RESULTS

Anthropometric, dry-land and swimming performance variables were similar between the groups at baseline (p > 0.05).

### Muscle strength

**FIG. 3:**
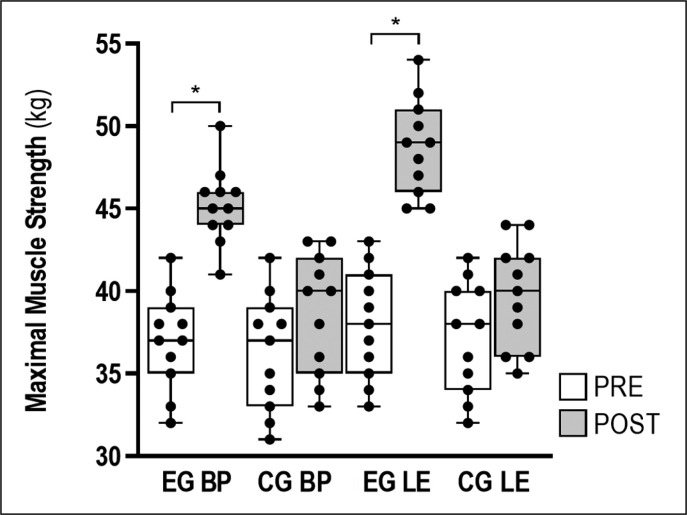
Box plots displaying the 25^th^ and 75^th^ percentiles, medians, whiskers extending from the minimal to maximal for maximum muscle strength (bench press, BP; and leg extension, LE) in both groups (CG and EG) after 8 weeks of training. (* *p* < 0.01)

Our results indicate that the 1-RM BP improved after 8 weeks in the EG (22.1%; 95% confidence interval [CI] 10.5 to 5.8 kg; p < 0.001; *d* = 3.25; very large). The 1-RM LE also increased after 8 weeks of combined training in the EG (28.2%; 95% confidence interval [CI] 13.5 to 7.9 kg; p < 0.001; *d* = 3.61; very large). Dry-land measurement remained unchanged in the CG (p > 0.05). The intraclass correlation coefficient of BP and LE test ranged between 0.81 (p < 0.01) and 0.72 (p < 0.01). All dry-land measurements can be observed in Table 3 and Fig. 3.

**TABLE 3:** Changes in swimming performance, technique and muscular strength after 8-weeks of training for both groups

Variables	Group	PRE	POST	p-value	Difference [95%CI]; (%Δ)	Effect size (d)
**Performance** (s)	**EG**	64.13 ± 1.41	61.85 ± 1.22	0.001	2.28 [1.10 to 3.45]; 3.55%	1.81, large
	**CG**	64.75 ± 1.55	64.29 ± 1.59	0.504	0.46 [-0.94 to 1.85]; 0.70%	0.02, trivial

**v_1_^st^_50-m_** (m · s^-1^)	**EG**	1.76 ± 0.12	1.94 ± 0.09	0.001	-0.18 [-0.27 to -0.09]; 10.17%	1.81, large
	**CG**	1.76 ± 0.12	1.79 ± 0.12	0.571	-0.03 [-0.14 to 0.08]; 1.65%	0.26, small

**SR_1_^st^_50-m_** (cycle · s^-1^)	**EG**	0.74 ± 0.04	0.80 ± 0.05	0.003	-0.06 [-0.10 to -0.03]; 8.65%	1.52, large
	**CG**	0.74 ± 0.04	0.74 ± 0.04	0.613	-0.01 [-0.04 to 0.03]; 1.35%	0.23, small

**SL_1_^st^_50-m_** (m)	**EG**	2.39 ± 0.04	2.43 ± 0.04	0.032	-0.04 [-0.07 to – 0.003]; 1.55%	1.03, moderate
	**CG**	2.38 ± 0.04	2.40 ± 0.03	0.461	-0.01 [-0.04 to 0.020]; 0.42%	0.03, trivial

**SI_1_^st^_50-m_** (m^2^ ·S^−1^)	**EG**	4.22 ± 0.34	4.71 ± 0.14	<0.001	-0.49 [-0.73 to -0.26]; 11.61%	1.96, large
	**CG**	4.20 ± 0.35	4.29 ± 0.34	0.547	-0.09 [-0.39 to 0.22]; 2.14%	0.27, small

**v_2_^nd^_50-m_** (m · s^-1^)	**EG**	1.73 ± 0.11	1.90 ± 0.13	0.003	-0.17 [-0.28 to -0.07]; 9.95%	1.52, large
	**CG**	1.73 ± 0.11	1.75 ± 0.11	0.683	-0.02 [-0.12 to 0.08]; 1.16%	0.19, trivial

**SR**_2_^nd^_50-m_ (cycle · s^-1^)	**EG**	0.72 ± 0.03	0.78 ± 0.05	0.003	-0.06 [-0.10 to -0.02]; 8.45%	1.53, large
	**CG**	0.72 ± 0.03	0.73 ± 0.04	0.462	-0.01 [-0.04 to 0.02]; 1.53%	0.33, small

**SL_2_^nd^_50-m_** (m)	**EG**	2.39 ± 0.05	2.43 ± 0.03	0.041	-0.04 [-0.07 to -0.002]; 1.51%	0.98, moderate
	**CG**	2.40 ± 0.05	2.39 ± 0.04	0.660	0.01 [-0.03 to 0.05]; 0.38%	0.20, small

**SI_2_^nd^_50-m_** (m^2^ ·S^−1^)	**EG**	4.13 ± 0.34	4.62 ± 0.34	0.004	-0.48 [-0.78 to -0.18]; 11.57%	1.47, large
	**CG**	4.15 ± 0.35	4.18 ± 0.33	0.831	-0.03 [-0.34 to 0.27]; 0.77%	0.09, trivial

**1-RM BP** (kg)	**EG**	37.00 ± 2.93	45.18 ± 2.32	<0.001	-8.18 [-10.53 to -5.83]; 22.11%	3.25, very large
	**CG**	36.27 ± 3.52	38.64 ± 3.64	0.137	-2.36 [-5.55 to 0.82]; 6.52%	0.69, moderate

**1-RM LE** (kg)	**EG**	38.00 ± 3.32	48.73 ± 2.90	<0.001	-10.73 [-13.50 to -7.96]; 28.23%	3.61, very large
	**CG**	37.18 ± 3.40	39.73 ± 3.20	0.086	-2.55 [-5.48 to 0.39]; 6.85%	0.81, moderate

*v:* swimming speed; SR: stroke rate; SL: stroke length; SI: stroke index; 1-RM: one maximum repetition; BP: bench press; LE: leg extension; EG: Experimental Group; CG: Control Group.

### Swimming performance and technique

Table 3 shows the swimming performance observed in both groups before and after 8 weeks; however, 100-m butterfly swimming performance improved at post-test only in the EG (3.5%; 95% confidence interval [CI] 1.1 to 3.4 s; p = 0.001; *d* = 1.81; large effect). The intraclass correlation coefficient of 100-m butterfly swimming performance was 0.91 (p = 0.01). Concerning technique, *v* improved at the 1^st^ 50 m and at the 2^nd^ 50 m in the EG (p < 0.01; *d* = 1.52 to 1.81; large effect). Likewise, SR increased at the two laps in the EG (p < 0.01; *d* = 1.52 to 1.53; large effect). SR (p < 0.05; *d* = 0.98 to 1.03; moderate effect) and SI (p < 0.01; *d* = 1.47 to 1.96; large effect) also improved at the two laps in the EG. Technique remained unchanged in the CG (p > 0.05). The intraclass correlation coefficient of all technique variables in the 1^st^ 50 m ranged between 0.83 (p < 0.01) and 0.89 (p < 0.01) and in the 2^nd^ 50 m ranged between 0.87 (p < 0.01) and 0.89 (p < 0.01).

## DISCUSSION

Training programmes display variable patterns of changes in strength, technique, and other energetic and anthropometric issues [1, 28], i.e. the training stimulus and subsequent adaptations constitute a dynamic and multifactorial process, and [2, 29]. Methodologies and phases in which training are offered to the swimmer can yield large differences in performance outcome [20, 28]. The main aim of our study was to evaluate the effect of combining HIIT and MST on muscular strength and butterfly swimming performance in competitive age-group swimmers. We observed significant improvements in maximum muscle strength, technique and 100-m butterfly swimming performance after 8 weeks.

### Effect of training on muscle strength

Combining HIIT and MST during 8 weeks improved maximum upper and lower body strength (1-RM BP = 22.1%; 1-RM LE = 28.2%). Girold et al. [32] reported that 12 weeks of MST improved elbow flexors in the isometric condition (39.5%) in competitive swimmers. Similarly, Girold et al. [5] found that 4 weeks of MST optimized the peak torque in the concentric condition at 60°· s^-1^ and at 180°· s^-1^ (11.2%, 16.9% respectively) in national level competitive swimmers. In other context, Amaro et al. [14] showed that training with 2–3 sets and 3–5 repetitions with intensity between 80 and 90% of 1-RM was beneficial to improve muscular strength. Recently, Kubo et al. [4] reported that 10 weeks of two different doses of MST, MST with higher load–lower repetition (4 repetitions · 7 sets) and MST with intermediate load–intermediate repetition (8 repetitions · 4 sets), improved the 1-RM BP (10 to 29.5%) in healthy men. Our study is in line with previous studies, linking dry-land training to swimming performance gain. The taper period is also essential for adaptation to stimuli, transferring strength gains to propulsive swimming actions [16]. Pritchard et al. [23] suggested that it is necessary to develop a taper period after an MST programme for maximal muscular strength gains. It must range from one to four weeks, be characterized by a decrease in the training load by 30–70% or reduced training frequency, preserving or slightly increasing intensity.

### Effect of training on technique and swimming performance

Swimming technique improved during the 1^st^ 50 m (1.55% to 11.61%) and 2^nd^ 50 m (1.51% to 11.57%). This improvement could be due to the improvement in maximum upper and lower body strength. However, Strass [21] found that 6 weeks of MST could improve the velocity of 25 m and 50 m in front crawl (1.3%; 2.1% respectively) in competitive swimmers. On the other hand, our results are in contrast with other studies that have shown that the HIIT programme does not improve kinematic variables in competitive swimmers [18–33]. The choice of the types of HIIT and the length of the intervention period in these studies could explain these different results.

The results of this study showed that 100-m butterfly swimming performance improved after 8 weeks with combined training (3.55%). In the same context, Ramos Veliz et al. [34] indicated that 18 weeks of combining training between dry-land training and HIIT could optimize the time of the 20-m maximal sprint swim (2.25%) in water polo players. Studies that integrate training in water regardless of HIIT or high-volume training (HVT) and dry-land workouts remain scarce [3, 16]. However, Pugliese et al. [35] observed that 6 weeks of a HIIT programme with 6000 m of HIIT · week^-1^ improved the 100-m front crawl (1.2%) in competitive swimmers. Moreover, Sperlish et al. [6] reported improvements in 2000-m front crawl (2.8%) after 5 weeks of a HIIT programme (30 min per session at 92% of personal best time) in healthy competitive young swimmers. The HIIT programme has shown beneficial results in the long distance performance [10, 36]. However, Rosenblat et al. 2019 stated that a workout programme containing 80% of HIIT volume could optimize endurance sport performance.

Concerning the relationship between MST and swimming performance, Keiner et al. [13] revealed that maximum strength in the bench press and squat explained 45–62% of the performance variance in 50- and 100-m swimming performance. Similarly, the effect of the MST programme on swimming performance has been tested by some studies [5, 8]. However, Girlod et al. [5] observed that the MST programme (3 sets · 6 repetitions at 80–90% of 1-RM) with exercises of press pull-up, draw with barbells, abdominal, squat and plyometric jumps, improved the 50-m front crawl performance (2%) in national-level competitive swimmers. Aspenes et al. [8] found that 11 weeks of the MST programme (3 sets · 5 repetitions) performing lat pulldown exercise did not improve the performance of 100-m front crawl in competitive swimmers. Despite some inconsistences in the literature, training intensity seems to be an important factor for performance improvements in swimming [20], and coaches should be (if they are not yet) aware about it.

However, it is important to acknowledge some shortcomings and potential limitations of our study. The current study is considered primarily applicable to a particular age group of male swimmers at a specific level of competition. Thus, it is necessary for more studies to cover female swimmers and other age categories. Future research in this area could also i) investigate the effects of various training protocols of MST with different doses of load volume (repetitions, sets, intensity); ii) investigate the effects of different types and lengths of a HIIT programme associated with swimming specificity; and iii) undertake integrated assessments of biomechanics and energetics (e.g. metabolic power, energy cost).

## CONCLUSIONS

Combining short-moderate HIIT (pool-based training) and MST in dry-land training workouts (1 or 2 sessions per week) is advantageous to enhance maximum muscle strength, technique, and 100-m butterfly swimming performance after 8 weeks in age-group swimmers. Although combining pool-based and dry-land workouts is a widespread practice in swimming training, our study provides evidence that combining short-moderate HIIT (pool-based training) and MST in dry-land training workouts is effective, highlighting its applicability for coaches and age-group swimmers. Adjusting training programmes accordingly can yield important improvements in swimming performance.

## Conflict of interest

The authors declare no conflict of interest.
